# Dental Caries, Periodontal Status, and Lifestyle Connections: Examining the Moderating Effects of Sleep, Smoking, Diet, and Mealtime Routines

**DOI:** 10.3390/nu17061079

**Published:** 2025-03-19

**Authors:** Marta Olmos-Valverde, María Carrillo-Díaz, María José González-Olmo, Martín Romero-Maroto, Isabel Jiménez-Trujillo

**Affiliations:** 1 Department of Orthodontics, Rey Juan Carlos University, Avda. Atenas s/n, 28922 Alcorcón, Spain; marta.olmos.valverde@urjc.es (M.O.-V.); mariajose.gonzalez@urjc.es (M.J.G.-O.); martin.romero@urjc.es (M.R.-M.); 2 Department of Paediatric Dentistry, Rey Juan Carlos University, Avda. Atenas s/n, 28922 Alcorcón, Spain; 3 Department of Preventive Medicine and Public Health, Rey Juan Carlos University, Avda. Atenas s/n, 28922 Alcorcón, Spain; isabel.jimenez@urjc.es

**Keywords:** oral health, healthy lifestyle, smoking, caries, mealtimes, sleep, balanced diet, students, periodontal status

## Abstract

**Background**: Lifestyle habits such as sleep, hygiene, or eating conducts are nowadays suffering from a lack of consistency, and this situation is being linked to systemic and mental health consequences. Nevertheless, not enough attention has been paid to investigate the plausible relation between lifestyle habits and oral health, and hence, this investigation aims to study the possible effects of certain lifestyle conducts on oral health in dental students. **Methods**: A sample of 195 dental students was gathered and basic sociodemographic data (gender, age, and nationality), hygiene habits, as well as data related to smoking, adherence to healthy habits, and daily schedule constancy were recorded. Oral health, with reference to decayed, missing, and filled teeth, was recorded using the DMFT index and bleeding upon probing (BOP). The Healthy Lifestyle Scale (EVS) was utilized to record sleep, smoking, and mealtime and diet characteristics. Descriptive analysis, Pearson correlations, a hierarchical linear regression model, and moderation analysis were performed. **Results:** The present evidence showed a direct relation between healthy lifestyle habits and oral health indicators. Respect to mealtime routines (MT), correct sleep hygiene habits (SR) and eating a balanced diet (BD) exert a moderator effect on caries and smoking. Smoking shows a positive correlation with the number of filled teeth and BOP. **Conclusions:** Findings suggest that healthy lifestyle habits are related to better oral health status. In addition, correct sleep hygiene habits, a balanced diet, and a mealtime schedule can act as a moderator factor between caries and smoking.

## 1. Introduction

The term healthy lifestyle refers to “a way of life that is based on the interaction between individual personal, social, socioeconomic and environmental living conditions” according to the WHO definition [1]. As stated by the same organization, the most prevalent oral diseases, such as caries and periodontal disease, would be included under the denomination of Non-communicable Diseases (NCDs), since although the bacteria responsible for them are transmissible, healthy lifestyle habits are considered a determining factor in their prevention [2].

Although there is no single agreed upon list of healthy lifestyle habits in the literature, it is true that most studies on the subject give greater relevance to several of them, such as never having smoked, frequent physical activity, correct stress management, a BMI of between 18.5 and 24.9 kg/m^2^, and appropriate dietary and sleep hygiene habits [3,4].

In reference to sleep hygiene habits, Hosker et al. define adequate sleep quantity as “that which allows the individual to feel rested and function optimally” [5]. The relationship between alterations in the quality and quantity of rest habits and general health has been widely analyzed in the literature, describing this relationship as bidirectional, since sleep can be affected by diseases such as diabetes, arterial hypertension, or cardiovascular diseases [6] and, in turn, lack of sleep could alter the functioning of the immune system and produce systemic inflammation [7].

There are also multiple connections between oral health and sleep. Classically, sleep disorders have been linked to alterations in the quantity and composition of saliva, highlighting alterations in immunoglobulin and interleukin values, as well as higher plaque and calculus values [7,8].

Regarding the relationship between caries and sleep, both Chen et al. and Ogawa et al. describe higher caries incidence in children with shorter total nighttime sleep duration and in general, worse consistency in bedtime schedules [7,9]. Dietary habits are known to be associated with various health problems, such as increased body mass index, metabolic changes, cancer, or immune system dysfunction [10].

In relation to diet, the literature defends not only the importance of its quality, but also of the frequency of intakes and their motivation (referring to the concept of emotional eating, which relates these aspects to the triggering of intakes due to emotional factors and not to hunger).

The relationship of these dietary patterns is also related to oral health, both directly and as a mediating factor. Different studies describe that a high fiber or vitamin D content would be related to a better periodontal status [11,12], or that healthy intake patterns result in better DMFT values [13].

It would, therefore, be important to assess the possible implications of subfactors such as the stability in behaviors related to these important aspects of oral health, which have not been taken into account individually until now, and which, being framed within factors such as diet and sleep, have not been the subject of research to date.

Oral implications of tobacco use are also well described in the literature, with oral cancer, periodontal disease, and caries being some of the most prevalent [14].

It is widely recognized that even though dental students should be well trained about oral health care, they can also be a vulnerable group due to unhealthy lifestyle habits due to their heavy workload, high levels of stress, and exhausting class schedules, despite being trained as health professionals. Therefore, the objectives of this research would be as follows:

- To evaluate the relationship between healthy lifestyle (smoking (SH), balanced diet (BD), respect for mealtimes (MR), and sleep hygiene (SR)) and oral health.
- To explore whether adherence to mealtimes, a balanced diet, and sleep hygiene could moderate the relationship between smoking and dental caries.

Based on these objectives, the hypothesis is that the above named healthy lifestyle habits, do have an impact on oral health. Therefore, proper adherence to a balanced diet, consistent mealtime routines, and correct sleep hygiene routines should be linked to a lower caries ratio and better periodontal health. Additionally, it is expected that healthy lifestyle habits will influence other lifestyle choices, such as smoking.

## 2. Materials and Methods

### 2.1. Study Design and Setting

A cross-sectional study was conducted at Rey Juan Carlos University in Alcorcón, Madrid, Spain. All dental students were requested to take part in the investigation, voluntarily, and without any extra compensation. The study received approval from the ethics committee of Universidad Rey Juan Carlos, under registration number ENM132/19 1111202020320.

Prior to beginning, the nature and aim of the research were explained to all students, who then signed an informed consent form. To ensure anonymity, students were instructed to identify themselves using the initials of their first and last name. Those with any local or systemic condition (such as disabilities, medication intake and/or preexisting pathologies) that could influence their oral health status, as well as those who declined to provide informed consent, were therefore excluded from the study.

The sample size was calculated by considering the number of dental students enrolled in the 2019/2020 and 2020/2021 academic years (N = 228), with an acceptable margin of error of 5% and a confidence level of 95%. Given these parameters, it was concluded that at least 136 students would be needed from the study population to ensure a statistically representative sample. Consequently, the sample of 144 students enrolled across all five years of the program was considered appropriate.

A specialist was asked to train two examiners, who were also calibrated, with the kappa statistic result during the calibration process being 0.91, suggesting a strong level of agreement.

Participants were organized into smaller groups and assembled in the waiting room of the Rey Juan Carlos University clinic, where they were provided with instructions to fill out the questionnaire, while an instructed member of the research team stayed in the room to address any questions they might have.

The oral health of the dental students was evaluated through clinical examinations. For this, a flat surface mirror, exploration and periodontal probes, gauze, and compressed air were used under artificial lighting conditions. The DMFT index, proposed by the World Health Organization (WHO), was calculated, referring to the number of decayed, missing, and filled teeth in permanent dentition, as well as the BOP.

### 2.2. Measures

As well as data on fundamental sociodemographic aspects (gender, nationality, age, etc.), crucial data on healthy lifestyle habits using a structured self-administered questionnaire were gathered. Furthermore, all participants underwent an oral examination to assess the DMFT index, which includes the total number of decayed (CD), filled (OD), and extracted teeth (ED), as well as the bleeding on probing index (BOP) [15,16].

To assess whether the participants maintain a healthy lifestyle, the *Estilo de Vida Saludable* scale (EVS) was utilized. This scale includes 12 items categorized into three subscales: eating habits, smoking habits, and sleep hygiene habits. Each item must be rated on a scale from 1 to 5 (where 1 means totally disagree and 5 means totally agree), and the total score from all items will indicate the subject’s lifestyle healthiness. Regarding a healthy diet, it allows us to find adherence not only to a balanced diet, but also provides information about meal scheduling. In relation to sleeping habits, it sheds light on resting times and quality and respect to sleeping time routines [17].

### 2.3. Statistical Analysis

Statistical analysis was conducted using SPSS version 27 (SPSS Inc., Chicago, IL, USA).

Descriptive analyses and Pearson correlations were carried out on the variables related to oral health, eating habits, and lifestyles, and hierarchical linear regression models were used to search for predictors of DMFT. Also, basic data analysis included descriptive statistics of sociodemographic variables (mean ± standard deviation) and a Kolmogorov–Smirnov test to assess the assumption of normality, which was confirmed, were performed.

Additionally, to explore whether lifestyle habits could have had moderating effects on the relationship between smoking and caries, a simple moderation analysis (model 1) was performed using the Hayes PROCESS module (version 3.3) for SPSS version 27, (SPSS Inc., Chicago, IL, USA).

Cronbach’s alpha was also calculated to assess the internal consistency of the instruments.

## 3. Results

### 3.1. Characteristics of the Sample

The sample included 195 subjects (50 males and 145 females), with a mean age of 24.5 ± 5.7 years old. The average and standard deviation for EVS, were 38.2 ±8, and 10.11 ± 3.24 for the SH subscale, 9.52 ± 2.92 for SR, 10.35 ± 2.53 for MR, and of 9.29 ± 2.52 for BD. The DMFT score was 4.5 ± 4.6, and the bleeding on probing (BOP) rate was 0.65%. Healthy gums, indicated by bleeding being less than 10%, were observed in 92.3% of the participants.

### 3.2. Relationship Between the Variables of Oral Health and Healthy Lifestyle Habits (EVS)

As can be seen in Table 1, healthy lifestyle habits (EVS) show a positive relationship with sleeping routines, adherence to mealtime schedules, and a balanced diet, reinforcing their protective role in oral health. These practices are negatively correlated with the DMFT index and the number of filled teeth, highlighting that a healthy lifestyle is associated with a lower burden of treated dental problems. In contrast, smoking habits (SH) show a weak correlation with most variables but a significant positive relationship with sleeping habits (SR, r = 0.221 *), filled teeth (r = −0.163 *) and bleeding on probing (BOP, r = −0.244), suggesting that smoking is a risk factor for gingival inflammation. Among oral health indicators, the DMFT index is strongly influenced by filled teeth (r = 0.912) and decayed teeth (r = 0.603). However, better lifestyle habits, including good sleep and regular mealtime schedules, are associated with lower DMFT values and its components (especially filled and decayed). Lastly, bleeding on probing (BOP) shows weak but significant correlations with indicators of poor oral health, such as filled teeth (r = 0.267). These results support the initial null hypothesis.

### 3.3. Moderation Analysis of Sleep Routines, Adherence to Mealtimes and Balanced Diet on Smoking and Dental Caries

A moderation analysis was performed using smoking as the independent variable, dental caries as the dependent variable, and adherence to sleep routines, mealtimes, and adherence to a balanced diet as moderating variables. As shown in Table 2, significant interaction values were obtained for all moderating variables: SR (β = −0.04, SE = 0.01, t = −3.71, *p* < 0.001, 95% CI [−0. 06, −0.02]), MR (β = −0.05, SE = 0.01, t = −3.46, *p* < 0.001, 95% CI [−0.07, −0.02]), and BD (β = −0.04, SE = 0.01, t = −2.48, *p* < 0.001, 95% CI [−0.07, −0.01]).

**Figure 1:**
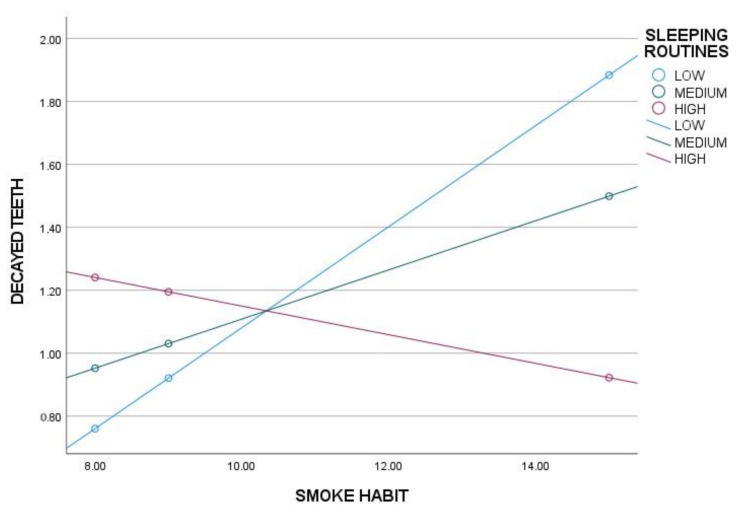
Moderation analysis of sleep hygiene habits (SR) on smoking habits (SH) and decayed teeth.

**Figure 2:**
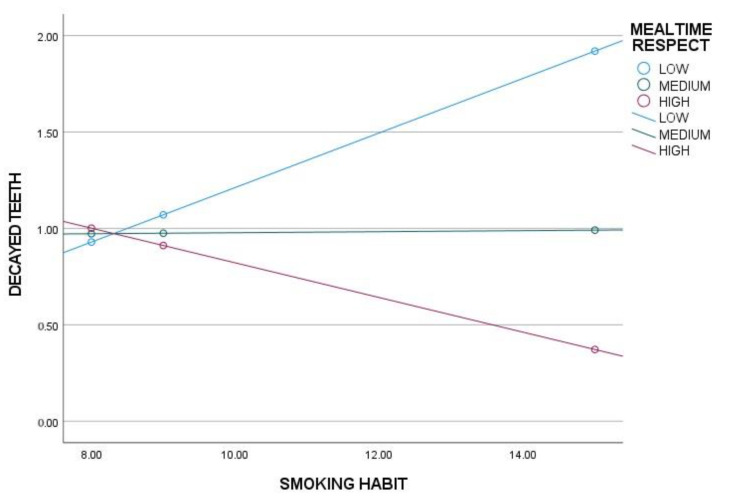
Moderation analysis of mealtime respect (MR) on smoking habits (SH) and decayed teeth.

**Figure 3:**
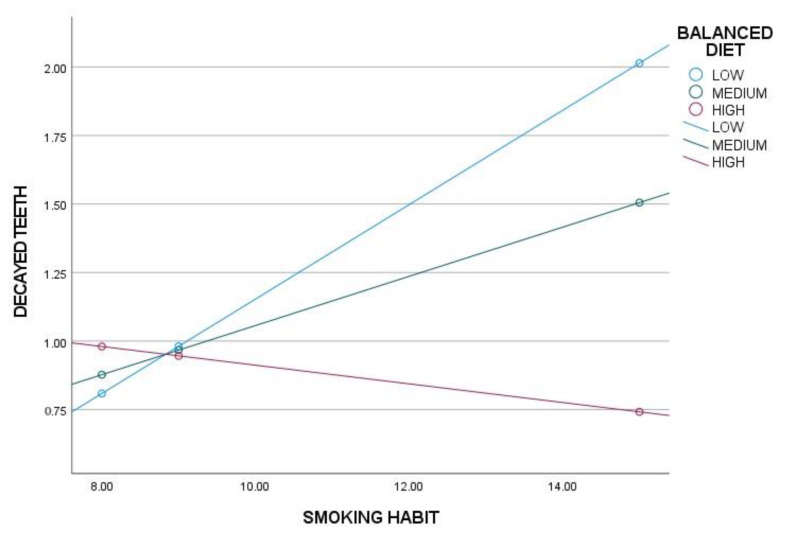
Moderation analysis of a balanced diet (BD) on smoking habits (SH) and decayed teeth.

To determine the conditions under which sleep routines (SR), respecting mealtimes (MR) and a balanced diet (BD) exert a moderating effect on the main model, significance levels and upper and lower bounds of confidence intervals were analyzed. The results indicate that sleep hygiene has a significant moderating effect only in individuals with low levels of SR, while no effect is observed in those with medium or high levels of SR (Table 3, Figure 1). Similarly, adherence to mealtimes shows a significant moderating effect only in individuals with low levels of MR, with no observable effect in those with medium or high levels of MR (Table 3, Figure 2). A balanced diet, on the other hand, exerts a significant moderating effect in individuals with low and medium levels of BD, but not in those with high levels of BD (Table 3, Figure 3).

### 3.4. Descriptive Studies of the Main Indicators of Dental Health in Students of the Degree in Dentistry at the Rey Juan Carlos University

As can be seen from Table 4, in the analyzed sample, it was found that the mean CAOD index value is 4.5, with a standard deviation of 4.6 (4.5 ± 4.6). On average, each student has 1 ± 1.7 untreated carious teeth and 0.21 ± 0.6 extracted teeth. Regarding gingival health, measured as the percentage of teeth showing bleeding after probing their gingival margins, the mean value obtained was 0.65%. Additionally, in 92.3% of the subjects analyzed, a gingival bleeding value of less than 10% of the teeth was found.

## 4. Discussion

The results of this study show that regarding diet, individuals who eat a balanced diet (understood as a diet capable of providing a state of general health and wellbeing), in which parameters are set as weekly intake of vegetables, fish, or legumes, as well as a global self-perceived dietary balance, showed better DMFT values [18].

These findings have been previously defended by other authors who maintain the influence of diet over oral health as a direct factor as well as a moderator factor [19].

In regards to the factor of respecting mealtimes as a habit, it has traditionally been considered an important factor towards dietary decision making, being linked to healthier choices, allowing a more regular range of intakes, or related to stable glucose blood levels. In fact, Chamorro et al. found in 2022 that individuals who adhere to a more stable mealtime schedule and in accordance with the body’s circadian rhythm would better facilitate its regulation [20]. Various studies also directly link poor adherence to mealtimes as a significant factor contributing to higher caries rates [21,22,23].

Some of the plausible causes that could explain the value of mealtime schedule stability as a predictor of oral health status have been previously named by other authors. Adherence to a mealtime schedule leads to choosing healthier options, moving away from “fast food” alternatives which have higher cariogenic potential [24]. It has also been previously described that individuals who skip breakfast, as a palpable way to measure lack to mealtime stability, tend to ingest more refined sugar, and it has also been linked to a higher rate of systemic inflammation, both situations which are associated to caries and gingival diseases [25,26].

Regarding sleep habits, that is, getting enough sleep to ensure rest while maintaining a stable bedtime, it is considered a factor that has a bidirectional effect on overall health, as it can affect hormonal regulation, including cortisol production and tolerance [27,28], and correlate with various diseases such as hypertension, metabolic disorders, depression, or cardiovascular diseases [29]. In addition, some conditions such as fibromyalgia, renal failure, or rheumatoid arthritis have been shown to cause sleep disorders [29].

With respect to the present investigation, data show that low adherence to SR leads to a lower scoring in oral health indicators (caries and gingival bleeding). These data are supported by previous studies, such as the study by Han et al., which reported poorer oral health in individuals who slept for shorter periods, or the study by Alawady et al., which reported that patients who slept less than 7 h per day had higher caries rates [30,31]. As in the current study, Shah et al. highlight the strong connection between sleep quality and quantity and oral health, also using caries and periodontal status as reference values [32].

It has also been argued that poor SR is associated with higher fat and bicarbonate intake and a tendency to eat between meals, which may be associated with a higher risk of caries [33].

Given the fact that meal timing consistency may have a moderating effect on the association between caries and smoking, a plausible explanation may be that people with poorer sleep quality tend to have a weaker immune response [34] and higher levels of salivary cytokines [35] or alterations in the normal oral microbiota [36], all of which pose risks that may increase the well-known harmful effects of smoking. In addition, the role of sleep hygiene as a moderator on the association between caries and smoking could be explained trough prior research that support changes in microbiota composition and saliva acidification in smokers [37], conditions which facilitate caries appearance, but that could be improved when sleeping quality is corrected, as defended by previous investigations that defend the key role of sleep and circadian rhythm in saliva production [38] and composition [39,40], improving the natural mechanisms of caries protection.

It is also well known that subjects who carry out a healthy balanced diet tend to show better oral health [41,42], but besides well-known direct action through the number and frequency of sugary intakes or pH levels [43,44], it could also act as a moderator factor for caries in smokers due to its influence over oral microbiome composition [45] which is usually altered in smokers [46]. A balanced diet has also been proven to act as a preventive factor against caries trough the ingestion of fruit, whole grains, and vegetables [47,48,49]. In the same vein, different authors have also linked gingival health status to healthy lifestyle habits [50], with smoking and an unbalanced diet being named as direct causes for severe gingivitis [51].

Regarding oral health status, the results of the present study indicate that the subjects have a mean CAOD value of 4.5, a figure very similar to that found in the research by Cortés et al. conducted on students from the University of Barcelona, who reported a CAOD value of 5.04. This is highly significant due to the cultural similarities between both populations, which should not only share scientific knowledge associated with the training received throughout the degree (which follows an almost identical curriculum) but also have similar social and cultural patterns [52].

Somewhat poorer results are described by Jouhar et al. (6.0 in their sample of Saudi students), although it is important to note that they only analyzed male students, a group to whom the literature historically assigns higher CAOD values [53].

Regarding data from previous studies analyzing gingival health in dental students, the results are consistent with those found in the present study, showing lower gingival bleeding than in the general population. However, most studies used indices other than BOP or, more commonly, self-perception scales, making comparisons more complex [54].

It is also important to point out some limitations. The validity of convenience sampling can be argued. In addition, the age of the participants is indicated within the range or average of similar studies [55,56,57]. From a gender perspective, there is an uneven distribution, as women tend to be highly represented in most health-related fields in our environment, and therefore, it would be important to take this inequality into account and further investigate on its possible repercussions in future research. Furthermore, for the mean values obtained for DMFT, the results are within the range found in previous oral health studies on dental students [58]. Another limitation stems from the use of self-reported measures, which may be affected by recall bias and social desirability-based responses. Furthermore, bitewing x-rays were not performed to confirm caries diagnoses or to assess fillings, along with the logical biases that it may imply. It would also have been desirable to consider other oral diseases and systemic interactions that can affect oral health, as shown in the literature by Pardo et al. [59].

Another factor to consider is the lack of follow-up over time, as this is an inherent limitation of cross-sectional studies.

Lastly, it would be important to extend this investigation to lay people, as dental students present generally better oral health status compared to the general population [48], where lifestyle habits hold implications over oral health that could be even more severe. It is also important to note that participants were not analyzed based on their year in the program. This was due to the complexity of determining each student’s year, as they could enroll in courses from different years if they had failed a key subject.

In view of the results, it would be advisable to establish a line of investigation that could infer these results to the general population and therefore establish educational policies to raise awareness of the importance of healthy lifestyle habits towards oral health. This information could be especially valuable in an already vulnerable group regarding oral health, such as smokers, who could be specifically beneficiaries of this information, and should be strengthened throughout the dental school curriculum.

It would also be useful to continue this line of investigation and further investigate how lifestyle habits can affect oral microbiome composition or saliva secretion.

## 5. Conclusions

Findings suggests that healthy lifestyle habits are related to better oral health status. In addition, correct sleep hygiene habits, a balanced diet, and adherence to mealtime schedules can act as moderator factors between caries and smoking.

## Figures and Tables

**Table 1:** Pearson’s correlation between the variables of oral health and healthy lifestyle habits (EVS).

	EVS	SR	SH	MR	BD	DMFT	Decayed	Filled	Missing	BOP
EVS	1									
SH	0.531 **	1								
SR	0.805 **	0.221 **	1							
MR	0.707 **	0.013	0.528 **	1						
BD	0.695 **	0.044	0.463 **	0.499 **	1					
DMFT	−0.282 **	−0.110	−0.233 **	−0.286 **	−0.160 *	1				
DECAYED	−0.098	0.034	−0.052	−0.169 *	−0.115	0.603 **	1			
FILLED	−0.285 **	−0.163 *	−0.246 **	−0.246 **	0.117	0.912 **	0.249 **	1		
MISSING	−0.073	0.087	−0.173 *	−0.173 *	−0.124	0.330 **	0.216 **	0.150 *	1	
BOP	−0.201 **	−0.244 **	−0.141 *	−0.141 *	0.193 **	0.193 **	−0.052	0.267 **	−0.037	1

Notes: EVS (healthy lifestyle habits), SH (smoking habit), SR (sleeping routines), MR (mealtime respect), BD (balanced diet), DMFT (decayed, missing, filled), BOP (bleeding on probing), * correlation is significant at the 0.05 level. ** Correlation is significant at the 0.01 level.

**Table 2:** Prospective prediction of adherence to sleep, mealtime routines, and a balanced diet on smoking and dental caries.

	*R* ^2^	*F*	*p*	Beta (SE)	*t*	*p*	95% CI
DV = Decayed teeth	0.07	4.94	<0.001				
Smoking Habit				0.45 (0.12)	3.73	<0.001	0.21, 0.69
Sleeping Routine				0.43 (0.13)	3.23	<0.001	0.17, 0.69
Interaction				−0.04 (0.01)	−3.71	<0.001	−0.06, −0.02
DV = Decayed teeth	0.09	6.02	<0.001				
Smoking Habit				0.51 (0.15)	3.45	<0.001	0.22, 0.80
Mealtime Respect				0.38 (0.15)	2.52	<0.001	0.08, 0.69
Interaction				−0.05 (0.01)	−3.46	<0.001	−0.07, −0.02
DV = Decayed teeth	0.05	3.68	<0.001				
Smoking Habit				0.46 (0.16)	2.89	<0.001	0.15, 0.78
Balanced Diet				0.36 (0.16)	2.22	0.03	0.04, 0.69
Interaction				−0.04 (0.01)	−2.48	<0.001	−0.07, −0.01

**Table 3:** Conditional effects of adherence to sleep routines, mealtimes, and a balanced diet on smoking and dental caries.

Sleeping Routine	Beta (SE)	*t*	*p*	95% CI
High	−0.05 (0.04)	−1.06	0.29	−0.13, 0.04
Medium	0.08 (0.04)	1.90	0.06	0.00, 0.16
Low	0.16 (0.05)	3.03	<0.001	0.06, 0.26
**Mealtime Respect**	**Beta (SE)**	***t***	***p***	**95% CI**
High	−0.09 (0.05)	−3.46	<0.001	−0.07, −0.02
Medium	0.38 (0.15)	2.52	<0.001	0.08, 0.69
Low	0.51 (0.15)	3.45	<0.001	−0.07, −0.02
**Balanced Diet**	**Beta (SE)**	***t***	***p***	**95% CI**
High	−0.03 (0.04)	−0.80	0.43	−0.12, 0.05
Medium	0.09 (0.05)	1.99	0.05	0.00, 0.18
Low	0.17 (0.07)	2.63	0.01	0.04, 0.30

**Table 4:** Descriptive studies of the main indicators of dental health in students of the Degree in Dentistry at the Rey Juan Carlos University.

	N	Min.	Max.	Media	Standard Deviation	IC 95%
Caries	195	0	8	1.03	1.74	(0.78–1.28)
Filled Teeth	195	0	20	3.28	3.67	(2.76–3.8)
Extracted Teeth	195	0	4	0.21	0.60	(0.13–0.29)
DMFT	195	0.00	28.00	4.52	4.62	(3.87–5.17)
BOP	195	0.00	9.26	0.65	1.85	(0.40–0.92)

Note: DMFT: DMFT index (carious, missing and filled teeth), BOP: bleeding on probing index.

## Data Availability

The data that support the findings of this study are available on request from the corresponding author. The data are not publicly available due to privacy and ethical restrictions.

## References

[1] Alpar S.E., Senturan L., Karabacak U., Sabuncu N. (2008). Change in the health promoting lifestyle behaviour of Turkish University nursing students from beginning to end of nurse training. Nurse Educ. Pract..

[2] Faught E.L., Gleddie D., Storey K.E., Davison C.M., Veugelers P.J. (2017). Healthy lifestyle behaviours are positively and independently associated with academic achievement: An analysis of self-reported data from a nationally representative sample of Canadian early adolescents. PLoS ONE.

[3] Li L.W., Wong H.M., McGrath C.P. (2017). Longitudinal Association between Obesity and Dental Caries in Adolescents. J. Pediatr..

[4] Ram S., Seirawan H., Kumar S.K., Clark G.T. (2010). Prevalence and impact of sleep disorders and sleep habits in the United States. Sleep Breath..

[5] Hosker D.K., Elkins R.M., Potter M.P. (2019). Promoting Mental Health and Wellness in Youth Through Physical Activity, Nutrition, and Sleep. Child. Adolesc. Psychiatr. Clin. N. Am..

[6] Nocini R., Favaloro E.J., Sanchis-Gomar F., Lippi G. (2020). Periodontitis, coronary heart disease and myocardial infarction: Treat one, benefit all. Blood Coagul. Fibrinolysis.

[7] Carra M.C., Schmitt A., Thomas F., Danchin N., Pannier B., Bouchard P. (2017). Sleep disorders and oral health: A cross-sectional study. Clin. Oral Investig..

[8] Leicht C.A., Goosey-Tolfrey V.L., Bishop N.C. (2018). Exercise intensity and its impact on relationships between salivary immunoglobulin A, saliva flow rate and plasma cortisol concentration. Eur. J. Appl. Physiol..

[9] Ogawa M., Ogi H., Nakamura D., Nakamura T., Izawa K.P. (2021). Association between Insufficient Sleep and Dental Caries among Preschoolers in Japan: A Cross-Sectional Multicentre Study. Eur. J. Investig. Health Psychol. Educ..

[10] Nielsen S.J., Trak-Fellermeier M.A., Joshipura K., Dye B.A. (2016). Dietary Fiber Intake Is Inversely Associated with Periodontal Disease among US Adults. J. Nutr..

[11] Altun E., Walther C., Borof K., Petersen E., Lieske B., Kasapoudis D., Jalilvand N., Beikler T., Jagemann B., Zyriax B.C. (2021). Association between Dietary Pattern and Periodontitis-A Cross-Sectional Study. Nutrients.

[12] Pfeiler T.M., Egloff B. (2020). Personality and eating habits revisited: Associations between the big five, food choices, and Body Mass Index in a representative Australian sample. Appetite.

[13] Marqués-Martínez L., Pérez-Bermejo M., Lairón-Peris A.R., Guinot-Barona C., Borrell-García C., García-Miralles E. (2022). Association between the Severity of Dental Caries and the Degree of Adherence to the Mediterranean Diet in the Pediatric Population. Nutrients.

[14] Ford P.J., Rich A.M. (2021). Tobacco Use and Oral Health. Addiction.

[15] Anaise J.Z. (1984). Measurement of dental caries experience—Modification of DMFT index. Community Dent. Oral Epidemiol..

[16] Shimazaki Y., Saito T., Kiyohara Y., Kato I., Kubo M., Iida M., Yamashita Y. (2006). The influence of current and former smoking on gingival bleeding: The Hisayama study. J. Periodontol..

[17] Leyton M., Lobato S., Batista M., Aspano M.I., Jiménez R. (2018). Validación del Cuestionario de Estilo de Vida Saludable (EVS) en una población española. Rev. Iberoam. Psicol. Ejerc. Deporte.

[18] Erdem R.Z., Bedir F. (2025). “Evaluation of the effect of nutrition and oral hygiene on Dmft index of patients applying to restorative dentistry clinic”. BMC Public Health.

[19] Isola G., Polizzi A., Alibrandi A., Williams R.C., Leonardi R. (2021). Independent impact of periodontitis and cardiovascular disease on elevated soluble urokinase-type plasminogen activator receptor (suPAR) levels. J. Periodontol..

[20] Chamorro R., Kannenberg S., Wilms B., Kleinerüschkamp C., Meyhöfer S., Park S.Q., Lehnert H., Oster H., Meyhöfer S.M. (2022). Meal Timing and Macronutrient Composition Modulate Human Metabolism and Reward-Related Drive to Eat. Nutrients.

[21] Dusseldorp E., Kamphuis M., Schuller A. (2015). Impact of lifestyle factors on caries experience in three different age groups: 9, 15, and 21-year-olds. Community Dent. Oral Epidemiol..

[22] Nagel G., Wabitsch M., Galm C., Berg S., Brandstetter S., Fritz M., Klenk J., Peter R., Prokopchuk D., Steiner R. (2009). Determinants of obesity in the Ulm research on metabolism, exercise and lifestyle in children (URMEL-ICE). Eur. J. Pediatr..

[23] van Loveren C. (2019). Sugar Restriction for Caries Prevention: Amount and Frequency. Which Is More Important?. Caries Res..

[24] Cândido A.C.O., Neves F.S., Fontes V.S., Melo A.S.T., de Faria E.R., Netto M.P., Oliveira R.M.S., Machado-Coelho G.L.L., Cândido A.P.C. (2023). Frequency of breakfast consumption and its associations with food consumption by degree of industrial processing and with indicators of overweight in Brazilian adolescents (EVA-JF Study). Nutrition.

[25] Fayet-Moore F., McConnell A., Cassettari T., Petocz P. (2019). Breakfast Choice Is Associated with Nutrient, Food Group and Discretionary Intakes in Australian Adults at Both Breakfast and the Rest of the Day. Nutrients.

[26] Muscente F., De Caterina R. (2020). Challenges in ischaemic heart disease: Not sleeping enough, not brushing your teeth, and skipping breakfast-three ways of increasing your risk of myocardial infarction?. Eur. Heart J. Suppl..

[27] Guyon A., Morselli L.L., Balbo M.L., Tasali E., Leproult R., L’Hermite-Balériaux M., Van Cauter E., Spiegel K. (2017). Effects of Insufficient Sleep on Pituitary-Adrenocortical Response to CRH Stimulation in Healthy Men. Sleep.

[28] Sejbuk M., Mirończuk-Chodakowska I., Witkowska A.M. (2022). Sleep Quality: A Narrative Review on Nutrition, Stimulants, and Physical Activity as Important Factors. Nutrients.

[29] Shapiro C.M., Devins G.M., Hussain M.R. (1993). ABC of sleep disorders. Sleep problems in patients with medical illness. BMJ.

[30] Han S., Jee D., Kang Y.J., Park Y.J., Cho J.H. (2021). Possible association between oral health and sleep duration: A cross-sectional study based on the Korean National Health and Nutrition Examination Surveys from 2010 to 2015. Medicine.

[31] Alawady A., Alharbi A., Alharbi H., Almesbah S., Alshammari N., Alkandari A., Alhazmi H., Alqaderi H. (2023). Association between sleep duration and dental caries in a nationally representative U.S. population. BMC Oral Health.

[32] Shah J., Poirier B.F., Hedges J., Jamieson L., Sethi S. (2024). Effect of sleep on oral health: A scoping review. Sleep Med. Rev..

[33] Zuraikat F.M., Wood R.A., Barragán R., St-Onge M.P. (2021). Sleep and Diet: Mounting Evidence of a Cyclical Relationship. Annu. Rev. Nutr..

[34] LaVoy E.C., Palmer C.A., So C., Alfano C.A. (2020). Bidirectional relationships between sleep and biomarkers of stress and immunity in youth. Int. J. Psychophysiol..

[35] Ibáñez-Del Valle V., Navarro-Martínez R., Ballestar-Tarin M.L., Cauli O. (2021). Salivary Inflammatory Molecules as Biomarkers of Sleep Alterations: A Scoping Review. Diagnostics.

[36] Park B., Koh H., Patatanian M., Reyes-Caballero H., Zhao N., Meinert J., Holbrook J.T., Leinbach L.I., Biswal S. (2023). The mediating roles of the oral microbiome in saliva and subgingival sites between e-cigarette smoking and gingival inflammation. BMC Microbiol..

[37] Santacroce L., Passarelli P.C., Azzolino D., Bottalico L., Charitos I.A., Cazzolla A.P., Colella M., Topi S., Godoy F.G., D’Addona A. (2023). Oral microbiota in human health and disease: A perspective. Exp. Biol. Med..

[38] Kurtović A., Talapko J., Bekić S., Škrlec I. (2023). The Relationship between Sleep, Chronotype, and Dental Caries-A Narrative Review. Clocks Sleep.

[39] Roestamadji R.I., Nastiti N.I., Surboyo M.D.C., Irmawati A. (2019). The Risk of Night Shift Workers to the Glucose Blood Levels, Saliva, and Dental Caries. Eur. J. Dent..

[40] Nishide S., Yoshihara T., Hongou H., Kanehira T., Yawaka Y. (2019). Daily life habits associated with eveningness lead to a higher prevalence of dental caries in children. J. Dent. Sci..

[41] Kilian M. (2018). The oral microbiome—Friend or foe?. Eur. J. Oral Sci..

[42] Sheiham A., James W.P. (2014). A new understanding of the relationship between sugars, dental caries and fluoride use: Implications for limits on sugars consumption. Public Health Nutr..

[43] Anderson A.C., Rothballer M., Altenburger M.J., Woelber J.P., Karygianni L., Lagkouvardos I., Hellwig E., Al-Ahmad A. (2018). In-vivo shift of the microbiota in oral biofilm in response to frequent sucrose consumption. Sci. Rep..

[44] Moynihan P. (2016). Sugars and Dental Caries: Evidence for Setting a Recommended Threshold for Intake. Adv. Nutr..

[45] Woelber J.P., Vach K. (2023). Healthier Smile: The Role of Diet and Nutrition in the Prevention and Therapy of Caries, Gingivitis, and Periodontitis. Nutrients.

[46] Belstrøm D., Holmstrup P., Nielsen C.H., Kirkby N., Twetman S., Heitmann B.L., Klepac-Ceraj V., Paster B.J., Fiehn N.E. (2014). Bacterial profiles of saliva in relation to diet, lifestyle factors, and socioeconomic status. J. Oral. Microbiol..

[47] Giacaman R.A. (2018). Sugars and beyond. The role of sugars and the other nutrients and their potential impact on caries. Oral Dis..

[48] Liu R.H. (2013). Health-promoting components of fruits and vegetables in the diet. Adv. Nutr..

[49] Ferrazzano G.F., Amato I., Ingenito A., Zarrelli A., Pinto G., Pollio A. (2011). Plant polyphenols and their anti-cariogenic properties: A review. Molecules.

[50] Olmos-Valverde M., Carrillo-Díaz M., González-Olmo M.J., Romero-Maroto M., Jiménez-Trujillo I. (2022). Evaluation of Dietary Habits, Type A Behavior Pattern and Its Relationship with Oral Health Status in Dental Undergraduate Students: A Cross-Sectional Study. J. Clin. Med..

[51] El Tantawi M., AlAgl A. (2018). Association between gingivitis severity and lifestyle habits in young Saudi Arabian males. East. Mediterr. Health J..

[52] Cortés-Martinicorena F.J., Ceballos L., Martínez-Pérez E., Hernández-Juyol M., Schulte A.G., Almerich-Silla J.M. (2022). Spanish Curriculum of Cariology Expert Group. Spanish Curriculum in Cariology for undergraduate dental students: Proceedings and consensus. Eur. J. Dent. Educ..

[53] Jouhar R., Ahmed M.A., Khurshid Z., Bokhari S.A.H. (2021). Association of BMI, Diet, Physical Activity, and Oral Hygiene Practices with DMFT Index of Male Dental Students at King Faisal University, Al-Ahsa. Nutrients.

[54] Rahman B., Kawas S.A. (2013). The relationship between dental health behavior, oral hygiene and gingival status of dental students in the United Arab Emirates. Eur. J. Dent..

[55] Dumitrescu A.L. (2007). Investigating the relationship between self-reported oral health status, oral health-related behaviors, type A behavior pattern, perceived stress and emotional intelligence. Rom. J. Intern. Med..

[56] Oancea R., Timar B., Papava I., Cristina B.A., Ilie A.C., Dehelean L. (2020). Influence of depression and self-steem on oral health-related quality of life in students. J. Int. Med. Res..

[57] Lin F., Ye Y., Ye S., Wang L., Du W., Yao L., Guo J. (2018). Effect of personality on oral health related quality of life in undergraduates. Angle. Orthod..

[58] Cortes F.J., Nevot C., Ramon J.M., Cuenca E. (2002). The evolution of dental health in dental students at the University of Barcelona. J. Dent. Educ..

[59] Pardo A., Barilli A., Signoriello A., Gualtieri M., Brancato G., Colapinto G., Lombardo G., Albanese M. (2024). Oral health conditions and hygiene procedures in patients with Parkinson’s disease: A systematic review. Explor. Med..

